# Fibrin monomer complex on postoperative day 1 is correlated with the volume of deep vein thrombosis after knee surgery

**DOI:** 10.1186/s40634-022-00482-y

**Published:** 2022-05-20

**Authors:** Manabu Akagawa, Hiroaki Kijima, Yoshiaki Kimura, Hidetomo Saito, Kimio Saito, Ikuko Wakabayashi, Takeshi Kashiwagura, Naohisa Miyakoshi

**Affiliations:** 1 Department of Orthopedic Surgery, Omagari Kousei Medical Center, 8-65 Omagari-torimachi, Daisen, Akita, 014-0027 Japan; 2 Department of Orthopedic Surgery, Akita University Graduate School of Medicine, 1-1-1 Hondo, Akita, 010-8543 Japan; 3 Department of Orthopedic Surgery, Akita City Hospital, 4-30 Kawamoto-Matsuokacho, Akita, 010-0933 Japan

**Keywords:** Deep vein thrombosis, Pulmonary embolism, Venous thromboembolism, Knee surgery, Thrombus volume, Fibrin monomer complex

## Abstract

**Purpose:**

Patients undergoing knee surgery are at high risk for deep vein thrombosis (DVT), which is infrequent but potentially life-threatening. It has not been identified how to efficiently detect high-risk DVT while minimizing bleeding complications from anticoagulation. We hypothesized that the degree of activation of thrombotic markers may correlate with the size of the thrombus. Therefore, we investigated the correlation between thrombotic markers and DVT thrombus volume in patients after knee surgery.

**Methods:**

This retrospective study involved 29 patients who underwent around knee osteotomy or total / unicompartmental knee arthroplasty from 2018 to 2020. Fibrin monomer complex (FMC) at 1, and 7 days after surgery, and D-dimer at 4, and 7 days after surgery were investigated. In addition, the volume of DVT was estimated with ultrasonography at the 7 days after surgery. Body mass index, surgical time, and total volume of blood loss were also evaluated. Factors related to thrombus volume were examined statistically.

**Results:**

Nine patients (31.0%) exhibited asymptomatic distal DVT, whereas 1 patient (3.4%) experienced asymptomatic proximal DVT. No patients had pulmonary embolism. Statistical analysis showed that only FMC concentration on postoperative day 1 was significantly correlated with thrombus volume (*p* <  0.001, 95% confidence interval 0.41 to 0.839, *r* = 0.679).

**Conclusions:**

The FMC concentration was a useful early indicator of deep vein thrombosis after knee surgery. Monitoring the FMC concentration could enable selective identification of patients with a high thrombus volume, which is associated with a high risk for pulmonary embolism.

## Background

Several surgical options are available for patients with knee osteoarthritis (OA), such as osteotomy, unicompartmental knee arthroplasty (UKA), and total knee arthroplasty (TKA). The choice of surgery is dictated by the degree of deformation, patient lifestyle and activity level. However, various complications have been reported with these surgeries, including venous thromboembolism (VTE), infections, fractures [1, 2], and loosening of the prosthesis [3]. Among them, VTE, which includes deep vein thrombosis (DVT) and pulmonary embolism (PE), is particularly dangerous and can be life threatening. Although deaths caused by VTE are rare, they still occur. Without thrombophylaxis, the rate of VTE after TKA is reportedly as high as 40–60% [4, 5]. In addition, recent investigations have reported that the rates of DVT after around knee osteotomy also occur ranging from 21.7–41% [6–8].

Due to the potential danger associated with DVT, many studies have examined approaches for its prevention and early detection. Fibrin-related markers such as D-dimer and soluble fibrin (SF) / fibrin monomer complex (FMC) are recognized as laboratory tests to assist in the detection of VTE [9]. A D-dimer value of 7.0 on postoperative day 4 or an SF value of 3.6 on postoperative day 1 have been determined as cut-off values for suggesting DVT in the leg after orthopaedic surgery [10]. Monitoring these fibrin-related markers is often combined with examination of the leg by ultrasonography (US), which is the first-choice imaging modality for DVT screening [11]. However, most thrombi detected during these examinations are distal DVT. For distal DVT that develops under prophylaxis, there is still no strong evidence for initiating or prolonging anticoagulation [12, 13]. It has also been reported that these screenings did not reduce the incidence of symptomatic VTE or fatal PE [14]. Furthermore, anticoagulation for the treatment of detected asymptomatic VTE has been reported to increase the risk of serious bleeding events [15]. For these reasons, several guidelines do not recommend postoperative routine screening for DVT [14, 16, 17]. Although this is partially due to the lack of evidence on the timing and methods of screening, currently there is no screening method that can lead to efficient and appropriate treatment. Nevertheless, we are always awaiting the advent of a screening method that can efficiently detect large, life-threatening VTE.

Since most PE which occurs after knee surgery originates from DVT, the size of the DVT may be related to the severity of PE. When a large thrombus is formed, significant activation of the coagulation/fibrinolysis system occurs. Therefore, we hypothesized that thrombotic markers may correlate with thrombus volume.

Hence, the correlation between thrombotic markers and DVT thrombus volume during the perioperative period in patients who underwent knee surgery was investigated in the present study.

## Methods

This retrospective study involved 29 patients who underwent around the knee osteotomy (AKO), TKA, UKA due to medial OA or spontaneous osteonecrosis of the knee from April 2018 to March 2020. A total of 12 men and 17 women were enrolled in the study; mean age of the patients was 68.5 years (range 54–83 years), the number of patients in each surgery were AKO:TKA:UKA = 16:9:4. AKO included medial open-wedge high tibial osteotomy, lateral closed-wedge high tibial osteotomy, distal femoral osteotomy, and the combination of femoral and tibial osteotomy (double-level osteotomy). Tourniquet was used in TKA / UKA. No patients had previous history of DVT or PE, and none of the patients received preoperative anticoagulant therapy. All surgeries were performed by 2 experienced orthopaedic surgeons. All patients received post operative physical prophylaxis. Postoperative anticoagulation (oral edoxaban) was given for 14 days only to TKA and UKA patients according to Japanese insurance indications.

Age, body mass index (BMI), surgical time, and total blood loss during the operation were recorded as the patients’ characteristics. In addition, FMC at 1, and 7 days after surgery (D1, D7), and D-dimer at 4, and 7 days after surgery (D4, D7) were measured.

**Fig. 1:**
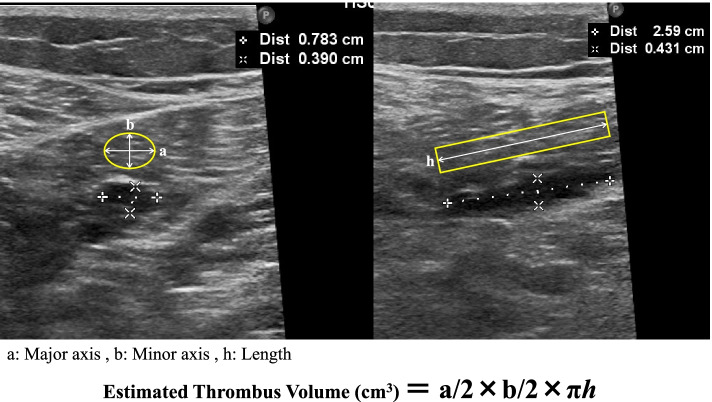
Method for estimation of thrombus volume. Thrombus volume was calculated by approximation of the value determined by ultrasonography to an elliptic cylinder [18]. a: Major Axis, b: Minor axis, h: Length. Estimated Thrombus Volume (cm3) = a/2 × b/2 × πh

Duplex doppler US investigations were employed to investigate for the presence of DVT at 7 days after surgery. DVTs were classified as proximal or distal depending on whether the thrombus was located above or below the popliteal vein, respectively. Thrombus volume was calculated by approximation to an elliptic cylinder based on the US measurement [18] (Fig. 1). If several thrombi were present, total thrombus volume was determined by summing respective volumes. Venographic computed tomography (CT) was performed only when proximal DVT was found or when patients experienced chest pain. Finally, the correlation between thrombus volume and age, BMI, operation time, total blood loss during surgery, FMC, and D-dimer was investigated.

All data were expressed as mean ± standard deviation (SD). The normal distribution of the evaluation items was tested by Kolmogorov-Smirnov test. Pearson correlation coefficient was used to examine the relationship between each evaluation items and thrombus volume. All statistical analyses were performed using EZR software [19], with *p* <  0.05 considered statistically significant.

## Results

Patients’ characteristics and investigated data are summarized in Table 1. Nine patients (31.0%) had asymptomatic distal DVT, and 1 patient (3.4%) had asymptomatic proximal DVT. In the patient with the proximal DVT, CT scanning confirmed that there was no PE. And none of the other patients had chest pain (Table 2). The incidence of DVT by procedure was 8/16 (50%) in AKO, 2/9 (22.2%) in TKA, 0/4 (0%) in UKA with proximal DVT occurring in TKA patient (Table 3). When compared AKO and arthroplasty patients, there was a tendency toward more DVT occurrence in AKO, but the difference was not significant (*p* = 0.06). Thrombus locations were as follows: common femoral vein, 1; posterior tibial vein, 3; peroneal vein, 1; soleus vein, 6 (Table 4). One patient had thrombus in both posterior tibial and peroneal vein. There was no complication other than DVT including infection, fracture, or systemic event during the perioperative period.

Kolmogorov-Smirnov test revealed that FMC D1 and D7 were non-normally distributed, but log-normally distributed. So, these data were log-transformed and used for statistical analysis. As a results of Pearson correlation coefficient, only FMC D1 was correlated with the thrombus volume (*p* < 0.001, 95% confidence interval 0.41 to 0.839, *r* = 0.679) (Table 5).

**Table 1:** Patient’ characteristics and results of the study

	Mean ± SD
Age (years)	68.5 ± 8.3
BMI (kg/m2)	24.9 ± 2.5
Operation Time (min)	129.6 ± 36.2
Total blood loss (ml)	38.4 ± 34.4
Sex (Male)	12
FMC D1 (μg/mL)	8.8 ± 10.2
FMC D7 (μg/mL)	7.7 ± 11.0
D-dimer D4 (μg/mL)	4.6 ± 3.0
D-dimer D7 (μg/mL)	8.3 ± 4.5
Total volume of thrombus (cm3)	0.7 ± 1.8

*FMC* Fibrin monomer complex

**Table 2:** Location of DVT in the study population (number and (%))

Distal DVT	9/29 (31.0%)
Proximal DVT	1/29 (3.4%)
Total	10/29 (34.5%)

*DVT* deep vein thrombosis

**Table 3:** The incidence of DVT by procedure (number (%))

	DVT
AKO	8/16 (50%)
TKA	2/9 (22.2%)
UKA	0/4 (0%)

*DVT* Deep vein thrombosis, *AKO* Arounf the knee osteotomy, *TKA* Total knee arthroplasty, *UKA* Unicompartmental knee arthroplasty

**Table 4:** Thrombus locations (number)

External iliac vein	0
Common femoral vein	1
Femoral vein	0
Popliteal vein	0
Anterior tibial vein	0
Posterior tibial vein	3
Peroneal vein	1
Soleal vein	6

**Table 5:** Results of Pearson correlation coefficient

	r	95%CI	*p* -value
Age	−0.31	−0.61 to 0.06	0.1
Total blood loss	0.21	−0.17 to 0.53	0.28
Body mass index	−0.03	−0.39 to 0.34	0.89
Surgical time	−0.005	−0.37 to 0.36	0.98
FMC D1	0.68	0.41 to 0.84	< 0.001^*^
FMC D7	0.08	−0.3 to − 0.43	0.68
D-dimer D4	0.25	−0.13 to 0.57	0.19
D-dimer D7	0.27	−0.11 to 0.58	0.16

*FMC* Fibrin monomer complex, *CI* Confidence Interval

*: Statistically significant (*p* -value < 0.05)

## Discussion

No markers have been found correlate with the size of post-operative DVT which can result in PE. In the present study, we revealed for the first time that the author know of, FMC D1 was strongly correlated with the volume of DVT thrombus in knee surgery patients. Additionally, routine anticoagulant prophylaxis tended to decrease the prevalence of DVT in patients of TKA/UKA compared to osteotomy patients. However proximal DVT was observed in TKA patient.

Previous report suggested that postoperative routine prophylaxis with anticoagulants has shown some efficacy [20]. However, even under the anticoagulant the prevalence of DVT is still 15% to 30% [21–23]. In this study, routine anticoagulant prophylaxis reduced the prevalence of DVT, but proximal DVT still occurred. Because of these facts, there are many reports on postoperative screening method of DVT, although severe PE after orthopaedic surgery is not frequent. Previously reported cut-off values for SF or D-dimer concentrations [9, 10, 24] are useful parameters for early identification of DVT. However, these were negative predictive cut-off values, and DVT identified using these cut-off values are often distal and of low volume. The problem is that, there is no consensus on whether new or extended anticoagulation therapy should be given for these distal DVTs. A recent systematic review showed extended prophylaxis has some efficacy, but concluded that benefit should be weighed against the risk of minor bleeding [25]. Japanese guideline of DVT recommended that, uniform anticoagulation of distal DVT detected by screening should be avoided [12]. So the indication for anticoagulation therapy should be determined based on risk-benefit considerations, but at present the criteria are not clear. Therefore, in our study, we examined factors that correlate with thrombus volume rather than with the presence of DVT. As a result, we found a strong positive correlation between thrombus volume and FMC concentration. A recent study revealed that thrombus volume is strongly correlated with PE severity [26]. So, with the result of present study, the risk of PE can be predicted immediately after surgery only by the blood sampling, and allow us selective intervention only to high risk DVT patients. The results may provide a useful decision criterion for the aforementioned problem with the treatment of distal DVT.

Furthermore, this selective intervention method may have cost-effective advantages. Recent systematic reviews showed that the combination of D-dimer and US are cost-effective method for the diagnosis of DVT [27, 28]. However, D-dimer is usually used to rule-out DVT, and if positive, it is not the criterion for selecting patients to be treated. On the other hand, our screening method, the combination of FMC D1 and US, allows us to select high-risk patients, which can be a criterion for selective intervention. If we can determine not only the presence of DVT, but also the selection of patients to intervene, it may be even more cost-effective than conventional methods of D-dimer and US.

However, the present study has several limitations. First, there is no cut-off value for the thrombus volume causing severe PE. The short-term mortality of PE is reported to be related to the right ventricle / left ventricle ratio as well as the thrombus volume [29], hence thrombus volume that can cause severe PE may vary depending on the patient’s body size. Therefore, the cut-off value for risk discrimination could not be determined. Further research will be necessary to determine the FMC cut-off value for the detection of large thrombus. Second, although US is suitable for DVT screening, it is not 100% sensitive. There are other evaluation methods such as three-dimensional US [30] or CT scanning that are more accurately detect and calculate thrombus volume. However, equipment capable of three-dimensional US is expensive, and CT has the problem of radiation exposure, so the application of these methods for daily screening is limited. We therefore believe that estimating thrombus volume using non-invasive two-dimensional US is more appropriate for clinical DVT screening. Another limitation is small number of patients. We would like to continue further study with more cases in order to improve statistical reliability and to determine cut-off value of FMC D1. Present research will be the first step toward more efficient prevention of PE.

## Conclusions

FMC concentration on postoperative day 1 was a useful early indicator of DVT volume after knee surgery. Monitoring FMC could enable selective identification of patients with a high thrombus volume, which is associated with a high risk for pulmonary embolism.

## Data Availability

The datasets used and/or analysed during the current study are available from the corresponding author on reasonable request.

## Abbreviations

OA: Osteoarthritis
TKA: Total knee arthroplasty
UKA: Unicompartmental knee arthroplasty
VTE: Venous thromboembolism
DVT: Deep vein thrombosis
PE: Pulmonary embolism
AKO: Around the knee osteotomy
SF: Soluble fibrin
FMC: Fibrin monomer complex
US: Ultrasonography
BMI: Body mass index
CT: Computed tomography
SD: Standard deviation
